# Compliance to Infection Prevention and Control Practices Among Healthcare Workers During COVID-19 Pandemic in Malaysia

**DOI:** 10.3389/fpubh.2022.878396

**Published:** 2022-07-18

**Authors:** Nadia Mohamad, Muhammad Alfatih Pahrol, Rafiza Shaharudin, Nik Khairol Reza Md Yazin, Yelmizaitun Osman, Haidar Rizal Toha, Normazura Mustapa, Zuraida Mohamed, Azyyati Mohammad, Rohaida Ismail

**Affiliations:** ^1^Environmental Health Research Centre, Institute for Medical Research, Ministry of Health Malaysia, Shah Alam, Malaysia; ^2^Disease Control Division, Ministry of Health Malaysia, Putrajaya, Malaysia; ^3^Occupational and Environmental Health Unit, Kelantan State Health Department, Ministry of Health Malaysia, Kota Bharu, Malaysia; ^4^Occupational and Environmental Health Unit, Johor State Health Department, Ministry of Health Malaysia, Johor Bahru, Malaysia; ^5^Occupational and Environmental Health Unit, Melaka State Health Department, Ministry of Health Malaysia, Melaka, Malaysia; ^6^Occupational and Environmental Health Unit, Negeri Sembilan State Health Department, Ministry of Health Malaysia, Seremban, Malaysia

**Keywords:** healthcare workers, infection control practices, risk, pandemic, COVID-19

## Abstract

Healthcare workers (HCWs) are at risk of contracting coronavirus disease-2019 (COVID-19) in their workplace. Infection prevention guidelines and standard operating procedures were introduced to reduce risk of exposure and prevent transmission. Safe practices during interaction with patients with COVID-19 are crucial for infection prevention and control (IPC). This study aimed to assess HCWs' compliance to IPC and to determine its association with sociodemographic and organizational factors. A cross-sectional study was conducted between March and April 2021 at public healthcare facilities in the east coast of Peninsular Malaysia. HCWs who were involved with COVID-19-related works were invited to participate in the online survey. The questionnaire was adapted from the World Health Organization (WHO) Interim Guidance: WHO Risk Assessment and Management of Exposure of Healthcare Workers in the Context of COVID-19. Respondents were categorized as compliant or non-compliant to IPC. A total of 600 HCWs involved in COVID-19-related works participated in the survey. Most of them (63.7%) were compliant to IPC as they responded to all items as “always, as recommended” during interaction with patients with COVID-19. The multivariate analysis showed that non-compliance was significantly associated with working in the emergency department (AOR = 3.16; 95% CI = 1.07–9.31), working as laboratory personnel (AOR = 15.13; 95% CI = 1.36–168.44), health attendant (AOR = 4.42; 95% CI = 1.74–11.24), and others (AOR = 3.63; 95% CI = 1.1–12.01), as well as work experience of more than 10 years (AOR = 4.71; 95% CI = 1.28–17.27). The odds of non-compliance among respondents without adequate new norms and personal protective equipment training were 2.02 (95% CI = 1.08–3.81) more than those with adequate training. Although most of the respondents complied to IPC protocols, compliance status differed according to department, work category, and years of service. Ensuring adequate training that will hopefully lead to behavioral change is crucial to prevent breach in IPC and thus minimize the risk of exposure to and transmission of COVID-19 in healthcare facilities.

## Introduction

The World Health Organization (WHO) declared coronavirus disease-2019 (COVID-19), which is caused by novel severe acute respiratory syndrome coronavirus 2 (SARS-CoV-2), a pandemic in March 2020. At that time, COVID-19 had spread rapidly in 114 countries with more than 118,000 confirmed cases, causing 4,291 deaths (1). After more than a year, the disease showed no sign of mitigation. Up until 6 July 2021, cumulative cases globally were more than 183 million with almost 4 million total deaths and over 2.6 million new cases being reported in a week (2). The overwhelming number of cases increased the burden for frontline healthcare workers (HCWs) in patient-facing roles and placed them at greater risk as their work require close contact with patients with COVID-19 (3).

The main mode of transmission for COVID-19 is human to human with respiratory droplets as the primary route of transmission. The SARS-CoV-2 route of entry to the respiratory systems are either *via* inhalation or deposition of droplets to mucous membrane or touching mucous membrane with SARS-CoV-2 contaminated objects (4). Available prevention guidelines on how to prevent COVID-19 transmission has remained unchanged from the early phase of the pandemic (4). Generally, physical distancing, face mask usage, frequent hand washing, good indoor ventilation, and avoidance of crowded places have been recommended (5). Additional implementation of personal protective equipment such as usage of gloves, gowns, face or eye protections and N95 masks, along with other standard practices, had been recommended for HCWs who are involved or in contact with patients with COVID-19 as part of infection prevention and control (IPC) during the pandemic (6, 7).

Despite the availability of infection prevention guidelines to protect HCWs, they are not immune to the disease. Previous evidence had shown that during the SARS-CoV-1 outbreak in 2003, a total of 1,706 HCWs were infected globally, contributing to 21% of total SARS cases (8). The current pandemic has shown a similar situation with HCWs comprising 14% of all reported cases (9). Nearly 570,000 HCWs in America were reported positive for COVID-19, and more than 2,500 of them were deceased by September 2020 (10). The WHO had estimated that the number of HCW deaths globally could be more than 115,000 within 18 months of COVID-19 emergence, and this was derived by population-based estimations (11).

By February 2021, Malaysia had recorded a total of 4,756 confirmed COVID-19 cases among HCWs prior to the national COVID-19 vaccination program (12). Despite preventive measures and completed 2 doses of vaccination, 2,341 confirmed COVID-19 cases were detected among HCWs within 3 months post-vaccination (13). Public healthcare system is the main healthcare provider in Malaysia, and the system is overwhelmed with the surge of cases during COVID-19 waves (14). Quarantine centers were established, and some government hospitals were redesigned into full or partial COVID-19 hospitals (15). Similar studies on compliance of HCWs to IPC during care of patients with COVID-19 and their associated factors have been carried out (16–22). However, they were confined mostly to HCWs working in hospitals. In Malaysia, management of and exposure to patients with COVID-19 involved HCWs from various types of healthcare facilities including hospitals, health clinics, and state and district health offices. The HCWs had different job scopes and level of exposure to or interaction with patients with COVID-19. Furthermore, there were limited studies that looked at the impact of organizational support to IPC compliance among HCWs. Thus, it is crucial to understand the role of organizational support and how exposure to SARS-CoV-2 and safe practices could reduce the risk of COVID-19 among HCWs in different types of healthcare facilities. A comparison of similar studies on compliance to IPC is available in **Supplementary Table S1**.

This study aimed to assess HCWs' compliance to IPC and to identify the associated sociodemographic and organizational factors that contributed to their compliance. The findings are expected to assist in investigating the trends of COVID-19 infection among HCWs and to assist in developing mitigation strategies to reduce COVID-19 transmission and protect our HCWs in their workplace. The tools from this study could be used by stakeholders in assessing adequacy of control and preventive measures among HCWs to other contagious outbreaks in the future.

## Materials and Methods

This was a cross-sectional study conducted at public healthcare facilities in a state in the east coast of Peninsular Malaysia involving 9 hospitals, 56 health clinics, and 10 district health offices. The online survey was emailed between March and April 2021 to all HCWs who were involved in COVID-19-related works including medical doctors, nurses, assistant medical officers, medical assistants, environmental health assistant officers, health attendants, laboratory personnel, and others (e.g., clerks, cleaners, and drivers). The survey link was sent through the occupational health unit of each facility. The link introduced briefly the study and approval that was obtained from the ethics committee and the state health department prior to commencement of this study. A detailed description of the study including objectives and participants' rights were explained in the first part after clicking the link, followed by informed consent. Respondents will be able to proceed to the questionnaire after providing their consent. A reminder for HCWs to fill up the questionnaire was sent by the occupational health unit at a 2-weeks interval throughout the 2-months study duration. Out of the 618 HCWs who responded to the questionnaire, 600 (97%) answered the questionnaire completely and met the criteria for involvement with COVID-19-related works. These included those who were directly involved in treating, managing or handling, and screening patients with COVID-19, conducting SAR-CoV-2 laboratory tests, transporting patients with COVID-19 and samples, cleaning COVID-19 facilities, and conducting epidemiological investigation on confirmed COVID-19 cases.

This study was approved by the Medical Research and Ethics Committee (MREC), Ministry of Health Malaysia [KKM/NIHSEC/P21–109(12)]. All participations were anonymous, and personal identifiers would not appear in any report.

### Study Tool and Variables

The questionnaire was adapted from the WHO Risk Assessment and Management of Exposure of Healthcare Workers in the Context of COVID-19 (23), which was structured in 4 parts. The first part was for gathering sociodemographic and occupational profiles consisting of variables such as age, gender, marital status, medical and medication history, workplace, job category, and years of service. The second part was about HCWs' activities related to COVID-19 exposure in the workplace and their COVID-19 status such as tested for COVID-19 and the result. The third part was about adherence to IPC during interaction with possible, probable or confirmed COVID-19 cases, which included assessment of PPE usage (5 items) and hand hygiene (4 items). Scoring for compliance status was similar to the WHO tool with a 4-point Likert scale: “always as recommended,” “most of the time,” “occasionally,” and “rarely.” While the terms used in this study for “high-risk exposure” were “noncompliance” and “low risk exposure” were identified as “compliance.” Those who responded to all items with “Always, as recommended” were categorized as compliant to IPC, whereas those with response other than that were categorized as non-compliant to IPC. Another modification was in scoring, which did not include adherence to IPC while doing aerosol-generating procedures. The last part was about organizational support. It consisted of 7 items to assess whether higher management in health facilities provided their workers with adequate instruments, items, training, or enforcement needed to ensure a safe work environment during the pandemic.

The survey forms were made available bilingually, in English and in Malay. The questions were translated into Malay language by 2 native Malaysians with good English proficiency, and back-translations were conducted by another two bilingual individuals to verify accuracy. The questions were modified according to local circumstances and were validated by five panels with occupational and public health background. Each panel indicated its comment or decision to remove, keep, or modify each item. After modification, content validation was conducted by another five public health specialists working at Ministry of Health's headquarters and the State Health Department. All of them were managing the occupational health program, including HCWs' well-being during the pandemic. Prior to the study, the questionnaire was tested on 50 HCWs in the Ministry of Health (MOH) who had an experience with COVID-19-related works. This was performed to ensure the readability, understanding and comprehensiveness of this tool and accuracy in reflecting the factors. The Cronbach's alpha was 0.748, which signified acceptable reliability.

### Data Analysis

Data from the questionnaires were transferred to Microsoft Excel, and Statistical Package for the Social Sciences (SPSS) version 21 (IBM, United States) was used for analysis. The data were initially analyzed descriptively to determine the representativeness of the respondents in this study. Categorical data were presented as frequencies and percentages, whereas means and standard deviations were expressed for continuous data. Pearson chi-square or Fisher's exact test was carried out to analyze activities with high exposure to SARS-CoV-2 and COVID-19 status with IPC compliance status. Next, univariate and multivariate analyses were conducted by binary logistic regression to identify a sociodemographic association with IPC as well as organizational support and IPC. Then, multicollinearity terms were checked, and the Hosmer-Lemeshow test and classification table were applied to check for model fitness. Statistically significant result was set at *p* < 0.05.

## Results

A total of 600 HCWs who were involved in COVID-19-related works were included in the survey. They were predominantly women (73.8%), married (90.3%), diploma or certificate holder (60.8%), without pre-existing medical condition (59.0%), and not on regular medication (75.0%). Mean age was 39.9 ± 7.4 years old, and mean household number was 5 ± 1.8. Nearly half of the respondents worked in hospitals (49.0%) and were nurses (52.0%). More than two-thirds of them had work experience of more than 10 years (69.5%) with mean work duration of 15.3 ± 7.3 years.

Table 1 shows the reported adherence to IPC practices. Adherence to type of PPE used and hand hygiene practices ranged from 83.7 to 97.5%; the highest adherence was for using medical masks and the lowest adherence was for using disposable gowns and single-use gloves. Overall, 382 (63.7%) of the respondents were compliant and adhered fully to all PPE and hand hygiene items (answered “always, as recommended”), making 218 (36.3%) of the respondents non-compliant.

**Table 1:** HCW adherence to infection prevention and control practices during interaction with patients with COVID-19.

**Variables**	**Always, as recommended *n* (%)**	**Most of the time** ***n* (%)**	**Occasionally *n* (%)**	**Rarely *n* (%)**
**PPE**
Single-use gloves	503 (83.8)	57 (9.5)	35 (5.8)	5 (0.8)
Medical mask	585 (97.5)	14 (2.3)	1 (0.2)	0 (0)
Face shield or goggles	523 (87.2)	53 (8.8)	21 (3.5)	3 (0.5)
Disposable gown	502 (83.7)	64 (10.7)	27 (4.5)	7 (1.2)
Remove and replace PPE as protocol*	539 (89.8)	48 (8.0)	10 (1.7)	3 (0.5)
**Hand hygiene**
After touching patient	565 (94.2)	33 (5.5)	0 (0)	2 (0.3)
Before and after clean or aseptic procedures performed	570 (95.0)	27 (4.5)	1 (0.2)	2 (0.3)
After exposure to body fluid	578 (96.3)	16 (2.7)	4 (0.7)	2 (0.3)
After touching patient's surrounding	537 (89.5)	57 (9.5)	5 (0.8)	1 (0.2)

^*^Remove and replace PPE as protocol—refer to the WHO interim guidance (e.g., when a medical mask became wet, dispose the wet PPE in the waste bin, perform hand hygiene, etc.).

Majority of the HCWs in this study provided direct care to patients (84.8%), but only 26.5% had face-to-face contact with patients with COVID-19, and 14.5% were present during aerosol-generating procedures (Table 2). Nearly two-thirds of the respondents (65%) had direct contact with contaminated objects or environmental exposure (bed, linen, medical equipment, bathroom, etc.) while caring for patients with COVID-19, and 2.8% were exposed to splash accidents (6 cases to eyes, 6 cases to mouth, and 10 cases to non-intact skin) and sharps injuries (2 cases) involving patients with COVID-19. However, no significant difference was found (*p* > 0.05) between their involvement in activities with high exposure to SARS-CoV-2 and compliance status.

**Table 2:** Activities with high exposure to SARS-CoV-2 and COVID-19 status according to IPC compliance status.

	**Compliance status**		
**Variables**	**Yes *n* (%)**	**No** ***n* (%)**	***p*-value^**a**^**
**Activitieswith high exposure to SARS-CoV-2**			
**Provide direct care to COVID-19 patients**			
Yes	327 (64.2)	182 (35.8)	0.487
No	55 (60.4)	36 (39.6)	
**Mobilized to carry out COVID-19 works**			
Yes	158 (61.7)	98 (38.3)	0.392
No	224 (65.1)	120 (34.9)	
**Face to face contact with COVID-19 patients**			
Yes	103 (64.8)	56 (35.2)	0.734
No	279 (63.3)	162 (36.7)	
**Direct contact with environment where COVID-19 patients were cared for**			
Yes	251 (64.4)	139 (35.6)	0.631
No	131 (62.4)	79 (37.6)	
**Present during aerosol-generating procedures**			
Yes	54 (62.1)	33 (37.9)	0.738
No	328 (63.9)	185 (36.1)	
**Involved in COVID-19 biological accident**			
Yes	9 (52.9)	8 (47.1)	0.351
No	373 (64.0)	210 (36.0)	
**HCW's COVID-19 status**			
**History of testing for COVID-19**			
Yes	178 (58.4)	127 (41.6)	0.006*
No	204 (69.2)	91 (30.8)	
**Positive by PCR for COVID-19**			
Yes	11 (84.6)	2 (15.4)	0.148^b^
No	371 (63.2)	216 (36.8)	

*^a^Pearson χ^2^ test*;

^b^Fisher's exact test; *p-value < 0.05.

Based on their COVID-19 status, Table 2 shows that out of 600 respondents, 305 (50.8%) had a history of taking a COVID-19 swab test either by procedural or asymptomatic screening or because they were in close contact to positive COVID-19 cases. Only 4.3% were positive for COVID-19. There was a significant difference in compliance status among respondents with history of swab testing, whereas compliance status was higher among those who had not undergone a swab test for COVID-19 (*p* = 0.006). However, there was no difference in compliance seen by positivity status to COVID-19.

Univariate and multivariate regression analysis were conducted to determine the association between sociodemographic and occupational factors, as well as organizational support and compliance status as shown in Tables 3, 4. The final model was checked for multicollinearity, and the variance inflation factor (VIF) for the variables was < 5, indicating no strong correlation between the variables. The Hosmer and Lemeshow tests were not significant (*p* > 0.05), which indicated that the model was fit. The overall correctly classified percentage is acceptable by the classification table.

**Table 3:** Demographic and occupational factors associated with compliance status among healthcare workers.

	**Compliance status**		**Univariate**		**Multivariate**	
**Variables**	**No *n* (%)**	**Yes** ***n* (%)**	**OR (CI = 95%)**	***p*-value**	**OR (CI = 95%)**	***p*-value**
**Gender**
Female	144 (32.5)	299 (67.5)	1		1	
Male	74 (47.1)	83 (52.9)	1.851 (1.277–2.683)	0.001*	0.830 (0.440–1.565)	0.565
**Workplace**
Hospital	87 (29.6)	207 (70.4)	1		1	
Health clinics	111 (40.7)	162 (59.3)	1.630 (1.151–2.309)	0.006*	1.663 (0.698–3.962)	0.251
District Health Office	20 (60.6)	13 (39.4)	3.660 (1.743–7.686)	0.001*	1.124 (0.196–6.441)	0.896
**Department**
Laboratory based	8 (40.0)	12 (60.0)	1.481 (0.450–4.876)	0.518	0.173 (0.013–2.298)	0.184
Medical based	22 (22.4)	76 (77.6)	0.643 (0.257–1.612)	0.347	1.154 (0.389–3.419)	0.797
Surgical based	13 (22.8)	44 (77.2)	0.657 (0.241–1.786)	0.410	1.195 (0.378–3.777)	0.762
Outpatient	87 (38.2)	141 (61.8)	1.371 (0.597–3.147)	0.456	1.447 (0.427–4.904)	0.553
Emergency	36 (50.0)	36 (50.0)	2.222 (0.892–5.534)	0.086	3.159 (1.072–9.312)	0.037*
Anesthesiology/ Intensive care	3 (14.3)	18 (85.7)	0.370 (0.087–1.585)	0.180	0.656 (0.137–3.131)	0.596
Public health	40 (53.3)	35 (46.7)	2.540 (1.024–6.298)	0.044*	1.598 (0.414–6.169)	0.497
Non-clinical based	9 (31.0)	20 (69.0)	1		1	
**Job description**
Nurse/ Midwife	84 (26.9)	228 (73.1)	1		1	
Medical Doctor	42 (39.6)	64 (60.4)	1.781 (1.121–2.829)	0.014*	1.148 (0.409–3.222)	0.794
Assistant Medical Officer	32 (43.8)	41 (56.2)	2.118 (1.252–3.584)	0.005*	1.957 (0.862–4.443)	0.108
Assistant Environmental Health Officer	20 (64.5)	11 (35.5)	4.935 (2.269–10.734)	<0.001*	5.352 (0.883–32.455)	0.068
Laboratory Personnel	11 (47.8)	12 (52.2)	2.488 (1.058–5.854)	0.037*	15.133 (1.360–168.438)	0.027*
Health attendant	19 (55.9)	15 (44.1)	3.438 (1.671–7.075)	0.001*	4.420 (1.738–11.242)	0.002*
Others	10 (47.6)	11 (52.4)	2.468 (1.011–6.022)	0.047*	3.632 (1.099–12.009)	0.034*
**Duration of employment**
Less 1 year	6 (30.0)	14 (70.0)	1		1	
1 to 10 years	62 (38.0)	101 (62.0)	1.432 (0.523–3.922)	0.484	2.505 (0.714–8.790)	0.152
More than 10 years	150 (36.0)	267 (64.0)	1.311 (0.493–3.482)	0.587	4.708 (1.283–17.274)	0.019*

*Only significant odds ratio was presented in Table 3*;

^*^p-value < 0.05.

**Table 4:** Organizational support provided by management in healthcare facilities.

**Variable**	**Compliance status**		**Univariate**		**Multivariate**	
	**Yes**	**No**	**OR (CI = 95%)**	***p*-value**	**OR (CI = 95%)**	***p*-value**
***Provide adequate temperature screening upon entering facility***
Yes	367 (64.7)	200 (35.3)	1		1	
No	15 (45.5)	18 (54.5)	2.202 (1.086–4.463)	0.029*	0.437 (0.138–1.385)	0.160
**Provide adequate hand washing facility or hand sanitizer**
Yes	377 (65.0)	203 (35.0)	1		1	
No	5 (25.0)	15 (75.0)	5.571 (1.996–15.550)	0.001*	2.470 (0.547–11.156)	0.240
***Provide adequate training for PPE and new norms***
Yes	351 (67.1)	172 (32.9)	1		1	
No	31 (40.3)	46 (59.7)	3.028 (1.854–4.946)	<0.001*	2.023 (1.075-3.809)	0.029*
**Enforce adequate wearing mask and physical distancing reminder**
Yes	375 (65.6)	197 (34.4)	1		1	
No	7 (25.0)	21 (75.0)	5.711 (2.386–13.666)	<0.001*	3.120 (1.000–9.729)	0.050
**Enforce adequate physical distancing markings (line, square, cross etc.)**
Yes	369 (65.4)	195 (34.6)	1		1	
No	13 (36.1)	23 (63.9)	3.348 (1.659–6.755)	0.001*	0.745 (0.250–2.220)	0.597
**Enforce adequate limitation the number of people in one area or room**
Yes	354 (65.8)	184 (34.2)	1		1	
No	28 (45.2)	34 (54.8)	2.336 (1.374–3.973)	0.002*	1.175 (0.536–2.574)	0.687
**Enforce at least 1 metre spacing between seats**
Yes	365 (65.9)	189 (34.1)	1		1	
No	17 (37.0)	29 (63.0)	3.294 (1.765–6.142)	<0.001*	1.648 (0.645–4.209)	0.296

^*^p-value < 0.05.

Age, educational level, number of households, preexisting medical condition, and taking regular medication showed no association with breach in IPC. There were five factors that were statistically significant for compliance status. Those who worked in the emergency department (AOR = 3.16; 95% CI = 1.07–9.31) had higher odds of non-compliance to IPC than those based in non-clinical departments. The odds of non-compliance to IPC were 15 times higher among laboratory personnel (AOR = 15.13; 95% CI = 1.36–168.44), 4.4 times higher among health attendants (AOR = 4.42; 95% CI = 1.74–11.24), and 3.6 times higher among other job categories (AOR = 3.63; 95% CI = 1.1–12.01) than nurses, whereas those who have a work experience of more than 10 years (AOR = 4.71; 95% CI = 1.28–17.27) had higher odds of non-compliance than those with < 1 year of work experience.

Table 4 describes the association between organizational support and compliance status among the respondents. It was found that the odds of non-compliance to IPC was 2 times higher among HCWs who lacked training than those who received adequate training. It was also found that the odds of non-compliance to IPC was 3 times higher if there were inadequate enforcement reminders for wearing a mask and physical distancing (*p* = 0.05).

## Discussion

The existing IPC standard in Malaysia is applied in healthcare settings to minimize the risk of infection for both patients and HCWs, and this is supported by the Occupational Safety and Health (OSH) program (24). During the pandemic, the Annex 21 Management of HCWs During the COVID-19 Pandemic has been developed and regularly updated to address standard operating procedures (SOP) (25). It includes awareness and training, IPC practices, PPE usage, vaccination, surveillance, and management of HCWs contracting the disease. The implementation of SOPs including IPC is regularly monitored and audited by the OSH or IPC committee in respective healthcare facilities.

Compliance status is important in identifying breach in IPC among HCWs. This is especially because since the start of the pandemic up to February 2021, more than half of infected HCWs in Malaysia contracted the disease at work (26). Preventing infections among HCWs is crucial to ensure there are no disruption of healthcare delivery during the pandemic. Staff shortage occurred not only because HCWs are positive and need to be isolated or treated but also because their colleagues become close contacts and need to be quarantined as well to prevent further transmission to others as mentioned before. In this study, 4.3% of respondents who underwent testing for COVID-19 were confirmed positive. This was consistent with findings from other studies in Italy (3.5%), Germany (3.5%), and the United States (4.5%) (27–29), while another review showed a higher percentage from HCWs tested by RT-PCR and detection of antibodies, with the pool prevalence of SARS-CoV-2 reported as 11 and 7%, respectively (30).

Compliance to IPC in other studies showed mixed findings from low to high practices (16–19). The majority of HCWs in this study showed good adherence to single items in IPC practices. Use of disposable gowns (83.7%) scored the lowest compliance among all personal protective equipment (PPE) used, while items under hand hygiene showed better results except for hand hygiene after touching patient's surrounding (89.5%), which was the only item that scored below 90%. The result was probably due to the illusion of safety, as there was no direct contact with patients. However, it is important to take precaution as the virus could also be transmitted from contaminated surfaces (31). In our study, there was no significant difference in compliance to IPC among HCWs based on their work during management of patients with COVID-19. Most of the respondents complied to IPC practices regardless of involvement in activities with high risk of exposure to SARS-CoV-2 or not. This is a commendable practice, as adherence to IPC is important in other daily activities, considering they can be exposed and contract COVID-19 infection even from the community (32). However, it is quite worrying that there was non-compliance to IPC practices even among HCWs who were involved in high-risk works, as they could get infected and increase the risk of nosocomial transmission to others (33).

The univariate analysis showed a significant association among status of compliance by gender, profession, type of facility and department where HCWs worked. Non-compliance was higher among men (47.1%) than women (32.5%), with the odds among men being 1.9 times higher than those among women. This could be contributed by their profession, as most of the women involved in the study were nurses, and they were also found to be more compliant than those with other types of profession in this study. Other studies also found that nurses were better in utilizing PPE than those with other professions (16, 20, 34, 35), while a seroconversion study in Egypt reported that the odds of hazard in women were 1.63 times higher than the odds in men (36). The medical doctors in this study had lower compliance than the nurses. Gilbert and Kerridge (37) reported reasons for lower compliance among medical doctors as they tend to rely on clinical judgment and experience rather than follow rules and ignorance, and some chose to disregard IPC practices despite recognizing their importance (37). Atnafie et al. (22) found that the rate of HCWs infected with COVID-19 among hospital staff was lower than that of HCWs working in other health facilities. However, they did not find any significant association (22). In our study, the odds of non-compliance were higher in HCWs working in health clinics and district health offices than in HCW working in hospitals. This is probably because hospitals have established IPC guidelines and have been practicing standard operating procedures on IPC even before the pandemic (24) compared to other types of health facilities. Moreover, infectious disease physicians and nurses are also posted in hospitals, and they have regular training and monitoring of IPC practices there (38). Similarly, HCWs who worked in public health departments had a significant association with non-compliance. This might be because common infectious diseases in community were tropical diseases like dengue and other diseases that are not spread by air or droplets, which have different protocols for IPC (39, 40).

After adjusting for other demographic and occupational factors, it was found working in emergency department (ED), worked for more than 10 years, HCWs who were laboratory personnel, health attendant and occupation grouped as others had significant risk of noncompliance. Non-compliance among HCWs in the ED could be contributed by the hectic and busy nature of work in the ED where there are many varied patients with different severity, with some requiring emergency procedures, making it difficult for them to keep changing their PPE each time for different patients (35, 41). A study by Ezike et al. found that preventive measures were not strictly adhered to in medical wards, children wards, and clinic and maternity complexes (21). The finding of significant non-compliance among HCWS who had worked for more than 10 years was consistent with the findings by Osborne (42). Greater non-compliance was found to be associated with longer years of working experience and habit as they could lead to disinclination to changes (42). However, our findings contradicted with another study in Canada that reported experienced nurses were more compliant than new nurses (43). Non-compliance was also seen among health attendants, laboratory personnel, and non-clinical staff compared to nurses. This category of HCWs usually does not have a direct contact with patients and this could probably influence their IPC practices. Nevertheless, they are still at risk, and IPC training should include them to improve their compliance (16).

Organizational support had been associated with compliance with using PPE in preventing respiratory diseases (43). This study demonstrated that all the organizational support provided had a significant association with compliance in the univariate analysis but after adjusting for confounders, only lack of adequate training was associated with non-compliance. Other studies had reported the importance of training and its influence on compliance with using PPE (44, 45). Inadequate training will lead to low knowledge of the importance and need for adherence to IPC among HCWs. Therefore, effective training in IPC should be endorsed to all medical staff (44) especially during this pandemic. Based on the findings, the questionnaire is able to assess IPC compliance among HCWs and would be useful to be incorporated in occupational health surveillance programs. Follow-up surveys should be carried out to observe whether there is improvement over time and to evaluate the effectiveness of intervention programs.

Among the limitations of this study was the use of self-administered questionnaire, which could lead to over- or under-reporting as compared to the real situation. Respondents will have to recall their practices when answering the question, which may contribute to recall bias. The IPC practices and compliance included in this study may also need to be revised in future studies with the emergence of new COVID-19 variants of concerns that are more transmissible (46–48).

## Conclusion

Generally, most of the HCWs in this study complied with IPC. The compliance status differed among HCW location, profession, and their years of service. However, it is a cause of concern that more than a quarter of the respondents were non-compliant to IPC practices during interactions with patients with COVID-19, which may expose them to SARS-CoV-2 infection in their workplace, especially when there are new emerging variants that are more transmissible. As this study has identified HCWs who are more likely to be less compliant, it is imperative that administrators of these health facilities look into ways to improve IPC compliance, which should include an infection control committee and an occupational safety and health committee. They could plan intervention programs to target non-compliant workers by sending reminders at regular intervals or conducting regular training, nudging strategies, and rewarding those who comply. They should also review the effectiveness of their intervention program by conducting regular monitoring of compliance.

## Data Availability Statement

The raw data supporting the conclusions of this article will be made available by the authors, without undue reservation.

## Ethics Statement

The studies involving human participants were reviewed and approved by Medical Research and Ethics Committee (MREC), Ministry of Health Malaysia [KKM/NIHSEC/P21-109(12)]. The patients/participants provided their written informed consent to participate in this study.

## Author Contributions

All the authors were involved in the conception and design of the investigation. YO, HT, NMu, ZM, and AM participated in the acquisition of the data. NMo, RS, and RI analyzed the data and interpreted the results. NMo, MP, RS, and RI wrote the manuscript, and all the other authors critically revised it. All the authors approved the final version of the manuscript. All authors contributed to the article and approved the submitted version.

## Conflict of Interest

The authors declare that the research was conducted in the absence of any commercial or financial relationships that could be construed as a potential conflict of interest.

## Publisher's Note

All claims expressed in this article are solely those of the authors and do not necessarily represent those of their affiliated organizations, or those of the publisher, the editors and the reviewers. Any product that may be evaluated in this article, or claim that may be made by its manufacturer, is not guaranteed or endorsed by the publisher.

## Abbreviations

COVID-19: coronavirus disease-2019
HCWs: healthcare workers
IPC: infection prevention and control
MOH: Ministry of Health
PPE: personal protective equipment
SARS-CoV-2: severe acute respiratory syndrome coronavirus 2
SPSS: Statistical Package for the Social Sciences
WHO: World Health Organization.
