# Supplementary material for: Compliance to Infection Prevention and Control Practices Among Healthcare Workers During COVID-19 Pandemic in Malaysia
Source: Front Public Health. 2022 Jul 18;10:878396. doi: 10.3389/fpubh.2022.878396 (PMC9340217; doi:10.3389/fpubh.2022.878396)
Supplement: Supplementary file 1 [file Table_1.DOCX]

Table S1: Compliance to Infection Control Practices among Healthcare Workers

| **Author** | **Methods** | **Findings** |
| --- | --- | --- |
| Ashinyo et al. (2021) | - Assessment of infection prevention and control compliance among exposed healthcare workers (HCWs) in COVID-19 treatment centers - Adopted questionnaire from the WHO Risk Assessment tool and IPC was analyzed as separate groups for hand hygiene and PPE use. | - Factors included sociodemographic, IPC facilities and occupation, but limited to working experience and profession - Univariate analysis showed non-clinical staff, secondary level education, HCWs who reported insufficient PPE were at risk of noncompliance. - Overall IPC was found to be high |
| Abed Alah et al. (2021 Nov) | - Assessment of compliance to PPE use and hand hygiene practice among HCWs in hospitals and clinics from the government, semi-government and private sectors. - Developed and adopted questionnaire from multi survey including the WHO Risk Assessment tool. IPC was analyzed as overall, hand hygiene and PPE use. | - Factors included sociodemographic, occupation, training and frequency of interaction with COVID-19 - Multivariate analysis showed higher IPC compliance among profession (dentist), health sectors (semi-governmental) and frequency of interaction with COVID-19 patients. - Overall IPC was found to be moderate. |
| Wong et al. (2021 Mar) | - Assessment of compliance to IPC and association between compliance and view on IPC among nurses in any healthcare settings from the government and private sectors. - Developed and adopted questionnaire from previous surveys including the WHO Standard Precaution in Healthcare and literature review. Standard precaution compliance was analyzed as overall | - Factors included sociodemographic, occupation (limited to employment status and service type) - Multivariate analysis reported working in designated team and pre-existing chronic conditions were associated with higher compliance in inpatient and high-risk group. While older aged respondents had high level of compliance among inpatient and outpatient groups. |
| Michel-Kabamba et al. (2020 Dec) | - Assessment of knowledge, practices, and PPE compliance among HCWs from government hospitals - Adopted questionnaire from the WHO Risk Assessment tool and IPC assessment was limited to PPE use only. | - Factors included sociodemographic, occupation and COVID-19 source of information - Knowledge, attitude and practices was analyzed using multivariate analysis - Majority of respondents did not comply with PPE use |
| Tsehay et al. (2021 Dec) | - Assessment of preventive practices among health care workers in a university hospital - Developed and adopted questionnaire from previous studies including the WHO COVID-19 Pandemic Preventive Practice | - Factors included sociodemographic, occupation and training - Multivariate analysis showed good preventive practices among nurses, female, working in the frontline and implementation of IPC guideline |
| Ezike et al. (2022 Jan) | - Assessment of compliance to risk perception, risk involvement/ exposure and compliance to preventive measures among nurses in a tertiary hospital - Adopted questionnaire from the WHO Risk Assessment tool and compliance analyzed separately for each IPC items | - Factors included sociodemographic and occupation. - Preventive measures were not strictly adhered in some departments (medical wards, children wards, clinic and maternity complex) |
| Atnafie et al. (2021 Apr) | - Assessment of exposure risks to COVID-19 among frontline health care workers in government hospitals and health centers - Adopted questionnaire from the WHO Risk Assessment tool and compliance analyzed separately for each IPC items | - Factors such as sociodemographic, occupation and IPC were analyzed for COVID-19 outcomes - Younger age groups, working experience (21-30 years) and good hand wash practices were protective against COVID-19 |
